# Integrity Testing of Pile Cover Using Distributed Fibre Optic Sensing

**DOI:** 10.3390/s17122949

**Published:** 2017-12-19

**Authors:** Yi Rui, Cedric Kechavarzi, Frank O’Leary, Chris Barker, Duncan Nicholson, Kenichi Soga

**Affiliations:** 1 Centre for Smart Infrastructure & Construction, Department of Engineering, University of Cambridge, Cambridge CB2 1PZ, UK; yr228@cam.ac.uk (Y.R.); ck209@cam.ac.uk (C.K.); 2 Arup Group Limited, London W1T 4BQ, UK; Frank.OLeary@arup.com (F.O.); Chris.Barker@arup.com (C.B.); Duncan.Nicholson@arup.com (D.N.); 3 Department of Civil and Environmental Engineering, University of California, Berkeley, CA 94720-1234, USA

**Keywords:** distributed fibre optic sensing, pile, heat transfer, integrity testing, finite element

## Abstract

The integrity of cast-in-place foundation piles is a major concern in geotechnical engineering. In this study, distributed fibre optic sensing (DFOS) cables, embedded in a pile during concreting, are used to measure the changes in concrete curing temperature profile to infer concrete cover thickness through modelling of heat transfer processes within the concrete and adjacent ground. A field trial was conducted at a high-rise building construction site in London during the construction of a 51 m long test pile. DFOS cables were attached to the reinforcement cage of the pile at four different axial directions to obtain distributed temperature change data along the pile. The monitoring data shows a clear development of concrete hydration temperature with time and the pattern of the change varies due to small changes in concrete cover. A one-dimensional axisymmetric heat transfer finite element (FE) model is used to estimate the pile geometry with depth by back analysing the DFOS data. The results show that the estimated pile diameter varies with depth in the range between 1.40 and 1.56 m for this instrumented pile. This average pile diameter profile compares well to that obtained with the standard Thermal Integrity Profiling (TIP) method. A parametric study is conducted to examine the sensitivity of concrete and soil thermal properties on estimating the pile geometry.

## 1. Introduction

Pile foundations have been in use for thousands of years [1] while concrete piles have become particularly widespread in the last 50 years. However, the growing use of larger diameter and longer piles has resulted in increased concern over the integrity and quality of cast-in-place foundation piles. This is compounded by the difficulty of inspection due to the large depth, limited accessibility and potential instability of shafts [2]. Moreover, the repair work of pile foundations is difficult and costly if large defects occur [3,4,5]. Therefore, testing techniques for the integrity of bored pile are highly valued. Traditional integrity testing methods includes cross-hole sonic logging (CSL), sonic echo (SE) testing, radiation based gamma-gamma logging (GGL), and, more recently, thermal integrity testing methods such as thermal integrity profiling or TIP [5,6,7,8,9,10].

Thermal integrity testing relies on measuring the amount of heat generated by a concrete element during curing. For a pile, the heat generated and its dissipation rate at a given location are a function of the concrete mix, the pile radius and the ground conditions. However, some defects such as poor quality concrete, necking, bulging, voids or soil inclusions will cause local abnormalities in temperature near the defect. Typically, an area in the concrete cover with temperature measurements lower than the overall cover average in the same ground layer would indicate a reduction in concrete volume and therefore a likely decrease in pile diameter. Inversely, higher temperature would indicate an increased concrete volume such as a bulge [11,12]. Hence, temperature measurements at regular depths intervals along the reinforcement cage and at various locations around its circumference provide thermal profiles from which the pile shape uniformity can be assessed by comparing the measured temperature to that expected for the concrete mix used, the given pile-soil boundary conditions and the ground thermal properties. Furthermore, the method can be used to assess cage concentricity within the concreted shaft since eccentricity would result in differences in temperature on opposite sides of the cage.

Distributed fibre optic sensing (DFOS) is gaining prominence in the field of structural health monitoring because of several advantages such as high spatial density of data, ease of installation and reliability. It has been used successfully in several applications such as monitoring the performance of ageing or new tunnels [13,14,15]; the behavior of thermal pile under coupled mechanical and thermal load [16,17,18,19]; displacement of diaphragm walls induced by excavation [20]; strain and crack of concrete pavement [21]; initiation and propagation of delamination of ultra-highperformance concrete overlay [22]; and is now regularly used as the preferred method for preliminary pile load tests [23].

This study proposes to build on the standard thermal integrity method, or TIP, through the use of distributed fibre optics sensing as an alternative to conventional thermal probes inserted through access tubes or embedded thermal sensors [10]. This approach has promising advantages over discrete instrumentation because it provides a high spatial density of data, where thousands of temperature points can be obtained along and around a curing concrete element, and because of the ease of installation of unobtrusive fibre optic cables and the very low failure rate of such sensors. In addition it is proposed that the data are analysed in conjunction with heat transfer process modelling taking into account the thermal properties of the ground strata in an attempt to improve the reliability of the thermal integrity testing method.

This paper presents a case study of a thermal integrity test carried out on a large bored concrete pile in London using a DFOS technique known as Brillouin Optical Time Domain Reflectometry (BOTDR). BOTDR was used to derive temperature profiles along the pile during concrete curing and the data were subsequently analysed by a one-dimensional axisymmetric heat transfer finite element model. It is shown that the pile diameter profile evaluated from the thermal data and heat transfer model is in relatively good agreement with conventional TIP results. A parametric study was conducted to examine the sensitivity of the concrete and soil thermal properties in estimating the pile profile using this DFOS-based thermal integrity testing technique.

## 2. Measuring Principle of the Fibre Optic Sensing Technique Used in This Study

The DFOS method used in this study is BOTDR, which is based on spontaneous Brillouin scattering. In general, it relies on the fact that when light travels through an optical fibre a small amount is backscattered due to small refractive-index or density fluctuations. The backscattered light spectrum has various components including the Brillouin frequency peaks, the position of which is sensitive to density changes caused by external factors such as strain and temperature.

Brillouin scattering arises from the interaction of the incident light wave photons with propagating density waves or acoustic phonons. These acoustic vibrations are generated by the thermal agitation of atoms in silica fibres and lead to density and refractive-index fluctuations. As long as the amount of light that is scattered by thermal fluctuations is too small to excite further fluctuations in the density of the medium, the process is known as spontaneous Brillouin scattering [24]. The scattering is inelastic and the photons may lose or gain energy (Stokes and anti-Stokes processes) and create or absorb phonons. This shift in photon energy corresponds to a shift in the frequency of the scattered light wave called Brillouin frequency shift. This shift is in the order of 10–11 GHz from the incident light wave frequency at a wavelength of 1550 nm. The value of this Brillouin peak frequency, ${ѵ}_{b}$, is proportional to the velocity of the acoustic phonons, $\upsilon_{a}$, and phase refractive index, n, which depend essentially on the local temperature and material density:

(1) $${ѵ}_{b}=\frac{2n\upsilon_{a}}{\lambda }$$

where λ is the wavelength of the incident light.

The relationship between this frequency and changes in longitudinal strain and temperature in the fibre core/cladding can be approximated by a linear function so that [25,26]:

(2) $$\Delta {ѵ}_{b}=C_{\varepsilon }\Delta \varepsilon +C_{T}\Delta T$$

where $\Delta {ѵ}_{b}$ is the change in Brillouin frequency due to a simultaneous change in strain, Δε, and in temperature, ΔT. $C_{\varepsilon }$ and $C_{T}$ are referred to as the strain and the temperature coefficient of the Brillouin frequency shift, respectively. This relationship is valid for a wide range of temperature and only deviates at very low or high temperatures [21,27].These coefficients depend on the material composition and geometry of the optical fibre and for standard telecommunication single mode fibres, used with BOTDR, $C_{\varepsilon }$ and $C_{T}$ will vary slightly at around values of 500 MHz/% and 1 MHz/°C, respectively, at the operating wavelength of 1550 nm. However, note that, once the optical fibre is packaged into a cable, this value of the strain coefficient can only be achieved if the strain applied to the cable jacket is fully transferred to the fibre through the cable layers. This need for good strain transfer within cables that are robust enough to survive the harsh environment of most industrial applications poses significant challenges in the design of specialist strain cables since standard telecommunication cables cannot be used. In any case, these cables should be calibrated within the range of strain expected for the application they are to be used for. This can be achieved by using strain rigs in which the cable is elongated by a known strain and by measuring the Brillouin frequency under constant temperature to derive the strain coefficient but also ensure that the relationship between strain and frequency is linear, with no hysteresis, and that the strain transfer is uniform along the cable.

In most applications, since variations in either temperature or strain can cause the Brillouin frequency to change, as in Equation (1), it is necessary to distinguish between these two effects, if one measures the Brillouin frequency change alone. One common solution to this problem, when using standard BOTDR, is to use a separate temperature compensation cable placed adjacent to the strain cable. This cable is commonly of a gel-filled loose tube construction where the optical fibre is isolated from mechanical strain effects. Hence, in this loose tube cable the frequency change is a linear function of the temperature variations only:

(3) $$\Delta {ѵ}_{b}=\beta \Delta T$$

where, β, is a constant obtained through temperature calibration representing a lump coefficient taking into account temperature effects as well as thermal expansion of the optical fibre in the loose tube [28]. Hence, temperature changes, ΔT, can be calculated independently and substituted into Equation (2) to obtain strain changes by measuring the central Brillouin frequency in both cables using a spectrum analyser as described below. The frequency in both cables can be measured simultaneously by splicing the cables into a single loop to avoid multiplexing through multiple channels. No strain data is used in this study and the strain data obtained during pile load test data is presented in details in Pelecanos et al. [23]. Hence, Equation (3) is used to solely calculate temperature changes during concrete curing at every sampling point to construct temperature profiles. The properties of the temperature cable used in this study are discussed in the next section.

BOTDR is a single ended technique where a light pulse is launched into one end of an optical fibre and the power of the spontaneous Brillouin backscattering is measured from the same end using a spectrum analyser. This power is measured in the time domain, by either heterodyne detection with a coherent receiver or optical frequency discrimination method by using a rejection filter to eliminate Rayleigh scattering signal [29]. In the heterodyne detection method, spectral filtering is achieved by mixing the backscattered light with an optical local oscillator before detection, or a microwave local oscillator after detection, followed by narrow band filtering. This allows a narrow spectral resolution so that one frequency component of the backscattered signal can be analysed at a time. The Brillouin spectrum can be reconstructed by changing the frequency of the local oscillator in successive increments.

**Figure 1:**
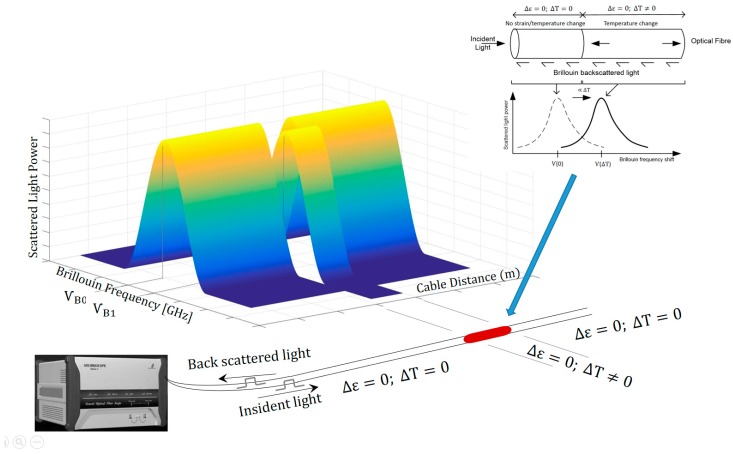
Brillouin gain spectrum and frequency shift caused by a change in temperature.

The amplitude of each frequency component is measured in the time domain and therefore the spatial position, z, from the where the pulsed light is launched to the position where scattered light is generated, can be determined using the following equation [30]:

(4) $$z=\frac{ct}{2n}$$

where c is the light velocity in a vacuum and t is the time interval between launching the pulsed light and receiving the scattered light at the end of the optical fibre. Figure 1 shows a schematic of the Brillouin gain spectrum and a frequency shift due to a change in temperature.

For each position, the frequency ${ѵ}_{b}$, corresponding to the peak power of the Brillouin spectrum (central frequency), can be determined by fitting the spectrum with an appropriate function such as a Lorentzian curve [31].

One important characteristic of distributed fibre optic sensing systems based on Brillouin scattering is the spatial resolution. The spatial resolution is the smallest distance over which strain or temperature can be measured with full accuracy. It is determined by the pulse width of the incident light. Narrowing the pulse width to improve resolution has limitations because a pulse width shorter than the phonon lifetime, which is approximately 10 ns in silica fibres, will lead to a broadened Brillouin gain spectrum, a weaker Brillouin signal and a sharp drop in measurement accuracy [24]. Hence the spatial resolution of most commercial BOTDR is limited to between 0.5 and 1 m [31]. Nevertheless data points can be sampled at intervals as low as a few centimetres by altering the sampling rate of the instrument digitizer. This so called sampling resolution is not a physical parameter and does not improve the spatial resolution but it can contribute to the spatial accuracy and the detection of physical events, notably sharp strain or temperature transitions. It is also worth noting that, although it is possible to carry measurements over several kilometres along a single optical fibre with BOTDR, larger pulse width and therefore lower spatial resolution is needed over such distances because of the loss in power due to increased attenuation.

A BOTDR was used in this study because it had been primarily specified to measure strain during load testing but provided a rare opportunity to gather temperature data during concrete curing for a large test pile. It is important, however, to stress that there are other distributed fibre optic measuring systems that can be used to obtain temperature distribution in concrete elements, often with greater precision and spatial resolution than BOTDR. This includes Brillouin scattering based double ended systems such as Brillouin Optical Time Domain Analysis (BOTDA) [22] or Raman scattering based systems known as distributed temperature sensing (DTS) systems [32,33]. Commercial DTS systems can only measure temperature but with higher accuracy and precision than Brillouin scattering based systems. They, however, suffer from complex calibrations procedures, which require corrections when optical losses occur through splices, connectors or macrobends [34].

## 3. Test Site and Instrumentation

### 3.1. Test Pile

The project considered in this paper is located on the Isle of Dogs in East London (UK). It consists of a 60 storey tall tower with a two level basement. Two London Underground (LU) running tunnels passing beneath the site about 13 m below the deepest excavation level, to which the building was allowed to transfer just minimal load resulting in a stiff piled raft solution being employed which transferred the building load to the large diameter piles either side of and between the tunnels. As the building load was effectively spanning the 11 m exclusion zone around the tunnel, column loads of up to 95 MN had to be accommodated. Such was the magnitude of the column loads that cast-in-situ bearing piles founded in the Chalk stratum with diameters up to 2.4 m and 61 m long were required. The novel nature of this pile type posed significant design and construction risk and therefore it was decided that a preliminary test pile was required to mitigate this risk.

**Figure 2:**
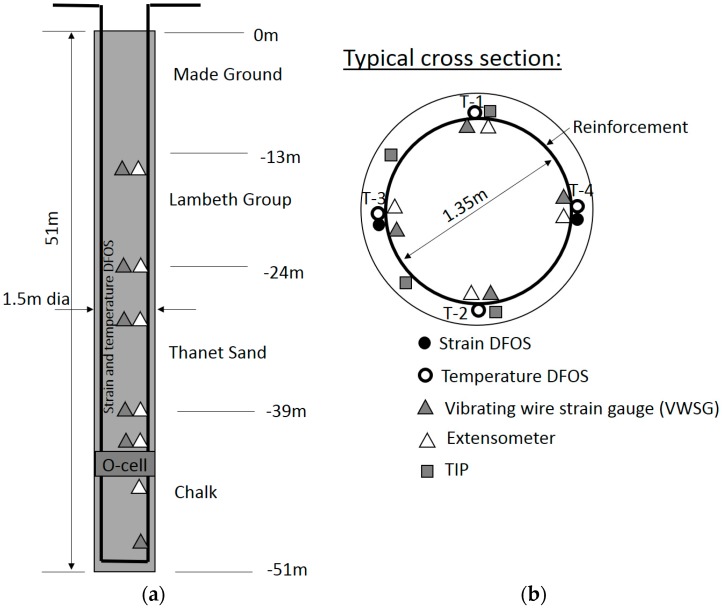
Geometry and instrumentation of the test pile: (**a**) plan view, (**b**) cross-section.

The test pile was constructed to closely match the methodology that would later be used for the working piles. As the working piles would be required to drill past the LU tunnels during engineering hours, when trains were no longer running and personnel could access the tunnels for monitoring purposes, it was decided to construct the test pile over two days. This therefore removed any necessity to reduce the shaft resistance parameters for the working piles due to the additional time required to construct the pile. The preliminary test pile was a 1.5 m diameter bored pile to a toe level of −44.5 mOD (50.9 m below ground level-bgl) and reinforcement cage diameter of 1350 mm, as shown in Figure 2a. The pile was constructed under bentonite support fluid. Due to the time required to construct the pile strict control was maintained over the bentonite properties. The reinforcement cage was assembled in four sections spliced together using couplers as they were lowered into the shaft. Construction of the pile took 47 h from first drilling below the permanent casing to completion of the placement of 90 $m^{3}$ of concrete. The top 13 m of superficial soil is made ground. It is underlain by Lambeth Group, with a thickness of 11 m, followed by 15 m of Thanet Sand. The lowest layer is chalk.

The pile was load tested using two No. 670 mm diameter bi-directional Osterberg load cells located around 6 m above the pile toe. The pile was tested using Osterberg cells due the large forces that were expected to be required to fail the pile (up to 87.5 MN). During the load test, the O-cells expanded bi-axially and reached a maximum force load of 30.9 MN in each direction (61.8 MN in total). The pile was monitored both during concrete curing and during subsequent load testing. The analysis in this paper focuses on the temperature data collected during concrete curing. The interpretation of the bi-directional O-Cell load test data is presented in Pelecanos et al. [23].

### 3.2. Pile Instrumentation

A BOTDR DFOS instrumentation scheme was installed alongside conventional thermal integrity testing sensors (TIP) and standard loading test instrumentation to measure temperature, strain and displacement during concrete curing and pile loading.

#### 3.2.1. Distributed Fibre Optic Sensing

**Figure 3:**
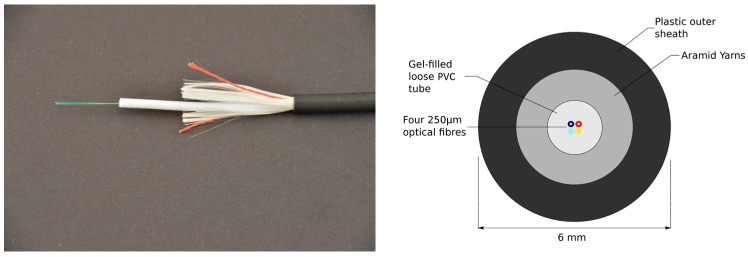
Construction details of the temperature cable (with permission from Kechavarzi et al. [28]).

Both strain and temperature single mode optical fibre cables to be used with a BOTDR analyser were installed on the pile. The strain cable, which consisted of a four-core reinforced ribbon cable manufactured by Fujikura (Tokyo, Japan), was used to measure strain during the load test and is not discussed further since this study focuses on temperature during concrete curing. A detailed description of the strain cable and its calibration is given in Kechavarzi et al. [28]. The temperature cable used in this study and shown in Figure 3 consisted of a standard gel-filled loose tube telecommunication cable distributed by Excel (Birmingham, UK). The 6 mm diameter cable was constructed of a PVC gel-filled loose tube hosting four 250 µm single mode fibres, surrounded by aramid yarns and a plastic outer sheath.

The cable was calibrated in the laboratory over a range of temperature representative of the conditions found on site to obtain the value of β, which could then be used in Equation (2) to calculate temperature changes from frequency shifts measured in the field. Approximately 10 m of cable was coiled loosely in a water bath (Grant Instruments Ltd., Shepreth, UK, model T100-ST18) and Brillouin central frequency changes measured using the BOTDR described below over the temperature range of 5–85 °C for one heating and cooling cycle. The overall relationship between temperature and frequency during the cycle was linear with coefficient of determination $R^{2}$ of 0.998 and β = 1.16 MHz/°C.

**Figure 4:**
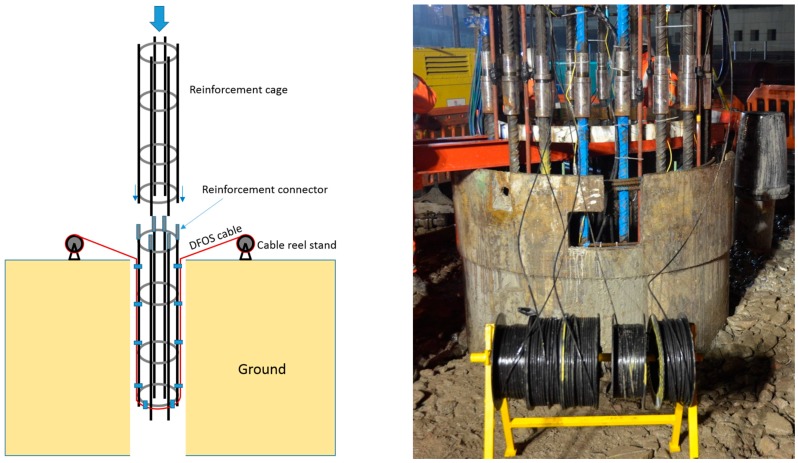
Lowering of reinforcement cages and installation of fibre optic cables.

The temperature cable sections used for analysis in this study consisted of two cable loops installed on four opposite sides along the pile. Their position is indicated in Figure 2b. The bottom section of the reinforcement cage was instrumented on the ground prior to lowering it into the drilled shaft. For each loop, half of the cable was pre-coiled onto two cable reels. The middle of the loop was first attached to the bottom link of the cage and then both cable reels uncoiled along the entire length of the bottom cage and attached to the rebars with cable ties. The cable reels with the remainder of the cables were secured to the top of the bottom cage. The bottom cage was lowered into the borehole and when the reels were reachable, they were removed from the cage and placed on reel stands positioned on both sides of the pile (Figure 4). Once the second cage section had been spliced to the bottom cage section, the cables were uncoiled from the reels and fastened tightly to the outside of the reinforcement cage using cable ties on reinforcement links as the cage was gradually lowered. The cable was sufficiently robust not to collapse under the pressure exerted by the ties and create unwanted restriction of the fibres. This installation process is shown in Figure 4.

The above procedure was repeated with the subsequent cage sections. Outside the pile, the cables were routed to a monitoring cabin where they were spliced together to form a continuous cable allowing for a single connection point to the spectrum analyser.

The spectrum analyser used in this study was a Neubrescope NBX-5000 BOTDR Analyser manufactured by Neubrex (Hyogo, Japan). The measurement precision or repeatability of the analyser (twice the standard deviation of the noise), for the measured distance and the spatial resolution used, was specified as ±2 °C. This repeatability or precision was tested in situ over two lower sections of approximately 40 m each for cables T-3 and T-4 one month after concrete curing. This assumed that the ground temperatures at depth lower than 10 m would not change significantly over a day. The precision obtained over 30 consecutive measurements was ±2.6 °C, which is close to the specified value obtained under ideal laboratory conditions. The spatial resolution, the minimum distance over which a change in temperature need to occur to be detected with full accuracy, was 0.5 m. The sampling resolution, the distance between two data points calculated from the time interval between two consecutive sampling points digitised by the instrument, was 0.05 m. This provided data points every 0.05 m (or a total of 4000 data points over the four different axial sides of the pile) though each of them was affected by temperature changes over the spatial resolution of 0.5 m. The acquisition time for each data set was 7 to 8 min, representing the time needed to average $2^{16}$ measurements in order to obtain the required precision. The analyser was operated continuously from the monitoring cabin within the specified temperature operating range of 10–35 °C.

#### 3.2.2. Other Instrumentation

In addition to the distributed fibre optics, the test pile was instrumented with vibrating wire strain gauges and extensometers. This supplemented the continuous strain data provided by the fibre optics along the pile depth. These data, which broadly agreed with that provided by the DFOS method, are presented in Pelecanos et al. [23] and are not discussed further in this paper.

The pile was also instrumented by the contractor with Thermal Integrity Profiling (TIP) wires, consisting of cables with thermistors installed in series, to test the integrity of the pile concrete. This method was chosen in preference to Crosshole Sonic logging to avoid the cage becoming overly congested with the required access tubes. The principle of the method is described briefly in Section 1. The cables with sensors were pre-attached to the reinforcement cage sections using cable ties and connected together when the cage sections were being spliced as the cage was lowered into the shaft. A total of six sensors arrays were as installed at various cross-sectional locations around the pile. Each array had sensors spaced 0.30 m apart starting at the top of the pile all the way down to the toe. Three of the arrays were partially faulty or did not record data for the first three days of curing and are not considered here. Following concrete pouring, the arrays we connected to data loggers and temperature recorded every 15 min. At specific times (usually 24 or 48 h after concrete placement) the temperature profiles are converted into shaft diameters at every measurement location based on the knowledge that temperature usually varies linearly with distance within the concrete cover (cover thickness) where the sensors are located. It is the understanding of the authors that this commercial method uses concrete logs to derive an average shaft diameter that, when compared with the average shaft temperature, provides a constant from which changes in temperature can be converted into changes in diameter [35].

## 4. Data Analysis and Interpretation

### 4.1. Field Data

The analysis in this paper focuses on temperature data collected during concrete curing. Concreting of the pile was carried out on 31 January 2014 between 09:25 and 14:20 by tremie placement. According to the concrete log, a total volume of 90 $m^{3}$ was casted. DFOS data collection was initialised at 15:48 with 7–8 min intervals between readings over a two-week period ending on 14 February 2014. The temperature at 15:48 was chosen as the baseline, the temperature data presented in this paper represent temperature changes relative to this baseline.

**Figure 5:**
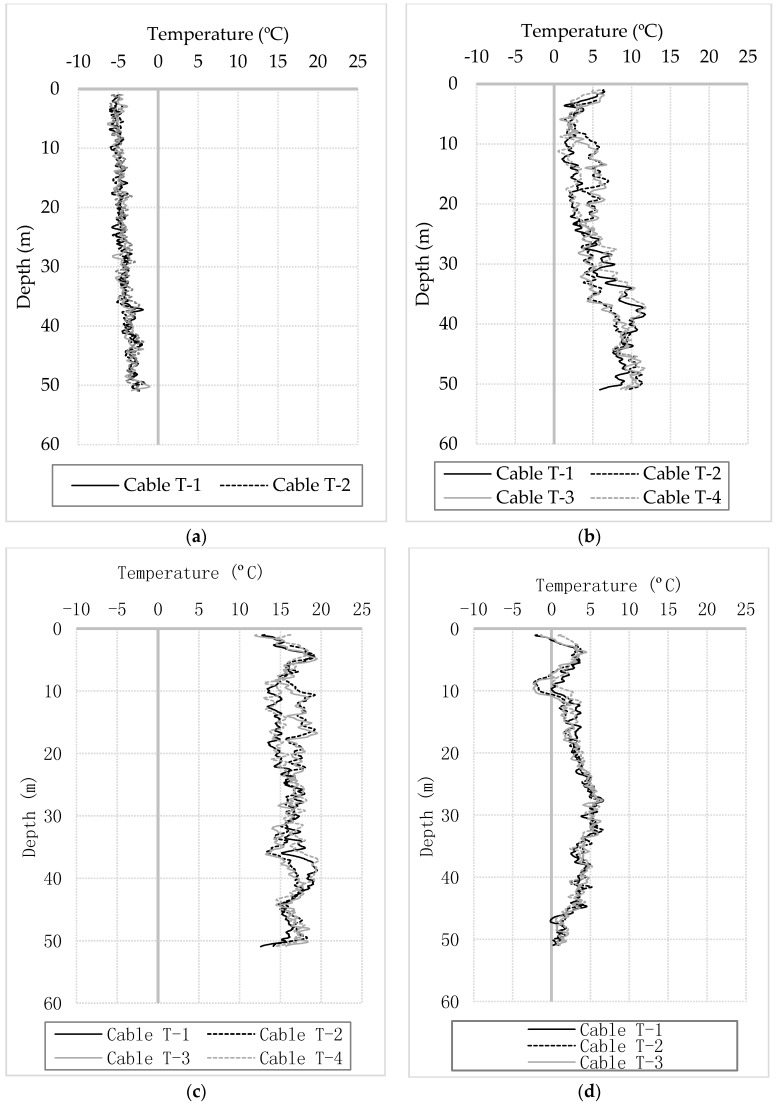
Longitudinal temperature profiles at (**a**) 4 h (**b**) 14 h (**c**) 1.4 days (**d**) 14 days.

Figure 5 shows temperature change profiles along the entire shaft length from the four cables at four stages. At the beginning, 4 h after the start of the measurements and therefore 5.3 h after concrete placement, the temperature had drops by about 2 to 5 °C along the shaft. This is possibly due to the temperature of concrete coming into equilibrium with the ground temperatures before the initiation of hydration and heat generation. The concrete mix was heavily retarded through the use of admixtures, which would have delayed the setting time of the concrete and thus the commencement of hydration. Between 4 and 14 h, the bottom of the pile started to heat up. The lower concrete was poured up to 5 h earlier than the top giving the hydration process some lead-time over the rest of the pile. At 1.4 days, the temperature profiles reach the maximum temperature values which had increased by about 14–20 °C compared to the baseline. Between 12 and 20 m, the temperature at locations of Cables T-4 and T-1 is about 2–4 °C lower than that at locations of cables T-3 and T-2. On the other hand, the trend is just opposite between 35 and 42 m. After that, the temperature reduces steadily towards initial temperatures. During the hydration process, the pile does not see a uniform temperature along its length, which indicates that the concrete volume is not uniform either.

**Figure 6:**
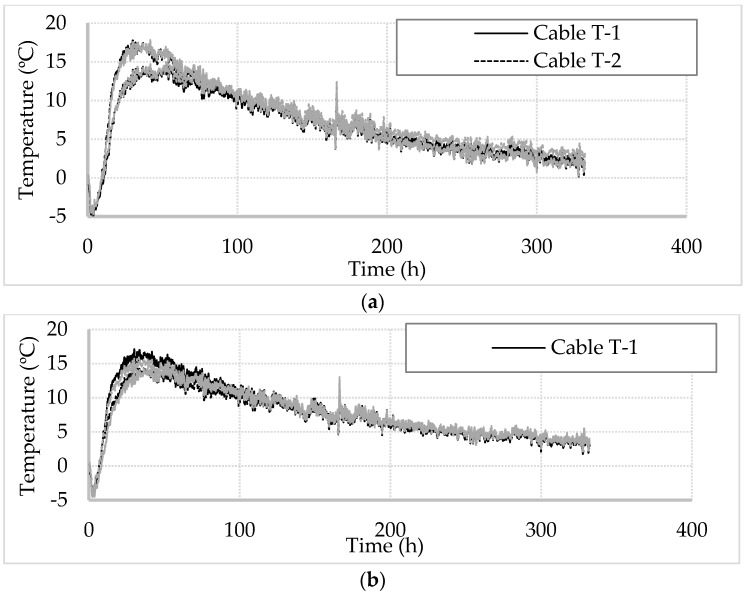
DFOS temperature development over time at two depths: (**a**) 15 m; (**b**) 35 m.

Figure 6 shows the change in temperature over the two-week monitoring period for two cross sections of the temperature cables at depths of 15 and 35 m. The observed trend showed a drop in temperature over the first two hours to about 4 °C below the initial temperature, as discussed earlier. The drop was followed by a steep increase in the temperature for the next 18 h after which the rate of heating began to drop. The maximum temperature at the depths shown was 17 °C warmer than the initial temperature and was observed between 30 and 40 h from the initial reading. The different peak temperatures between the cables in each plot show that the pile did not see a uniform maximum temperature on each cross section. The difference ranged between 3 and 5 °C despite the rate of heating being similar across the cables. Closer to the top of the pile, the T-1/T-4 side is cooler. However, at lower depths the T-1/T-4 side is warmer. Steady cooling began after about 40 h with the temperature gradually reducing for the remainder of the test. At the end of the two-week monitoring period the temperature of the pile at these two locations was close to 5 °C above the initial condition and had not reached equilibrium.

### 4.2. Finite Element Model

**Figure 7:**
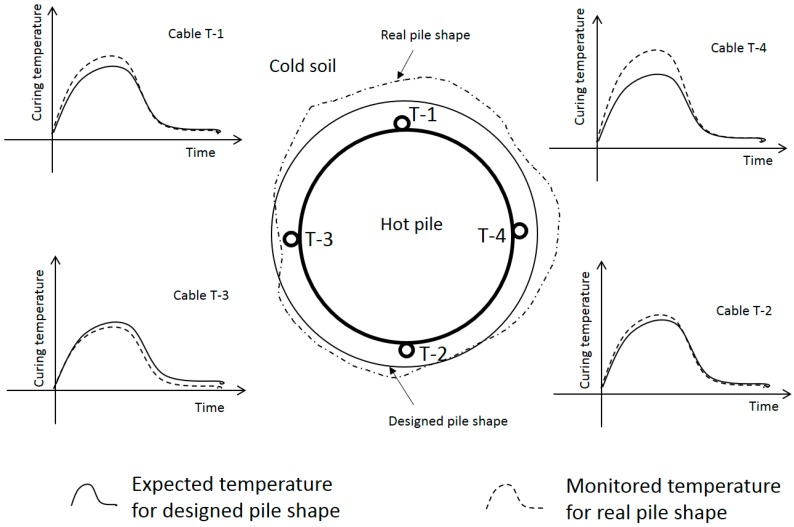
Conceptual relationship between DFOS temperature and pile radius on a typical cross section.

**Figure 8:**
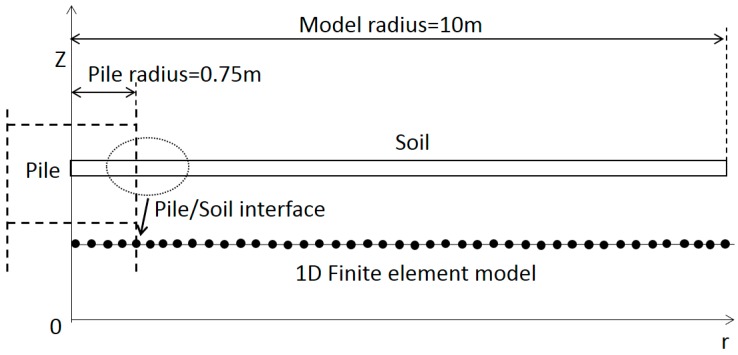
1D axisymmetric heat transfer finite element model.

As described above, there are four temperature readings on every cross sections at 0.05 m intervals along the four FO cables on the shaft length and therefore close to 4000 data sets of temperature-time curves. Each temperature profile is dependent on the combination of factors of hydration heat and the heat transfer between the pile and soil, as shown in Figure 7. These temperature curves can be computed by a thermal finite element (FE) analysis. If the temperature curve from the FE analysis can match the DFOS data, it indicates that the assumed pile geometry in the FE model is close to the real situation. If not, by adjusting cover thickness (position of the pile/soil interface as shown in Figure 8), the FE model can be optimised by dichotomy to better match the DFOS temperature data. Using this method, the cover thickness can be predicted at four locations on every cross sections at 0.05 m intervals along the whole pile.

During thermal integrity testing, heat conduction is the major form of heat transfer. The fundamental law governing heat conduction for this problem is the first law of thermodynamics, commonly referred to as the principle of conservation of energy. The pile length is very large in comparison with the dimensions of the structure in the other two horizontal directions. Hence, away from the pile ends, the heat transfer is assumed to happen in the horizontal direction only. On the other hand, the thermal boundary condition at pile ends would have influence on the thermal behaviour of pile during the curing stage. As can be seen in Figure 5c, as the temperature of hydration reaches its maximum, large temperature gradients have developed at the upper and lower boundaries. These are larger at the upper boundary because of the cold winter air temperature. This heat transfer influence in the vertical direction violates the assumption of 1D axisymmetric heat transfer in the horizontal direction. This influence is significant within approximately one diameter of the top and bottom of the deep foundation pile [10]. Hence, the FE analysis was conducted on the dataset between 2 and 49 m depth.

In addition, due to the axial symmetry, the finite element model for thermal integrity testing can be simplified as a 1D model, as shown in Figure 8. This assumption was confirmed by conducting a 3D FE analysis for selected pile geometries (results not shown). The FE model includes pile element and soil element, and hydration heat source is applied on every nodes of the pile elements to simulate the hydration heat production. The pile/soil interface location (or concrete cover thickness) is adjusted in the FE analysis to match the predicted temperature change with the DFOS data.

Hydration heat plays a crucial role in the temperature development of early-age concrete [36,37]. It has significant effects on material properties, and hence influences the life-time performance of concrete. Prosen et al. [38] use isothermal microcalorimeter to study early hydration reactions during the hydration of cement. Following Prosen et al.’s experiment, Bentz [39] developed a three-dimensional hydration and microstructure model for Portland cement. However, they did not propose explicit formulas of hydration heat production. On the other hand, De Schutter and Taerve [40,41] developed a classical general hydration model based on the results of their isothermal and adiabatic hydration tests. In this hydration model, the heat production rate is expressed as a function of the actual temperature and the degree of hydration. Pane and Hansen [42] modified De Schutter’s method for blended cements. Moreover, they demonstrated a similar trend between the degree of reaction and bound water content. The work of Gruyaert et al. [43] also indicates a good match between reaction degrees and the hydration degrees determined by BSE-image analysis. Another model of concrete hydration heat is developed in an engineering software High Performance Paving Software (HIPERPAV) of the Federal Highway Administration (FHWA) [44]. Similar to the model by De Schutter [40], this hydration heat production model is also a function of temperature and the degree of hydration [37,44,45]. The results show that the HIPERPAV temperature model produced accurate predictions of the in-place temperature development of hydrating concrete.

In this study, the hydration heat model by De Schutter [40] is used to interpret the temperature profiles during the curing stage to assess the pile geometry. The thermal analysis needs to be performed for four sides on every cross section. The model has explicit and simple mathematical expression. The heat evolution of cement is obtained by the superposition of the heat productions of the Portland reaction. The evolution of mechanical properties in early-age cement is described using functions of the degree of hydration [40]:

(5) $$P\left(t\right)=q_{\max,20}\cdot c\cdot {\left[\sin\left(\alpha_{t}\pi \right)\right]}^{a}\cdot e^{-b\alpha_{t}}\cdot e^{-\left[\frac{E}{R}\left(\frac{1}{T_{c}}-\frac{1}{T_{r}}\right)\right]}$$

where *a*, *b* and *c* are the material constants controlling the distribution of hydration heat production; $\alpha_{t}$ is the degree of reaction, defined as the fraction of the heat of hydration that has been released; E is the apparent activation energy of the P-reaction, R is the universal gas constant, $q_{\max,20}$ is the maximum heat production rate of the S-reaction at 20 °C, $T_{c}$ is the temperature of concrete (K), and $T_{r}$ is the reference temperature (293 K).

Table 1 shows the concrete components used to construct the test pile. According to De Schutter [40], the cement (Type 1) is the only source of hydration heat in this type of concrete, which takes up about 13% by weight. Hence, the total released heat $Q_{\max}$ and maximum heat production rate $q_{\max,20}$ equals 13% of the values by pure cement (Type 1). The other parameters are the same as those derived from experimental data by De Schutter [40], as listed in Table 2.

**Figure 9:**
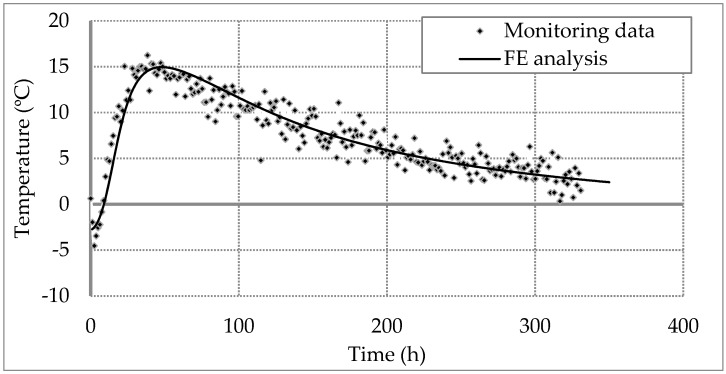
Calibration of finite element model.

A 1D axisymmetric finite element model with total radial length of 10 m is developed. Table 3 lists the thermal parameters of the soil used in this finite element model. All these parameters are selected from the reasonable range by Garber [46], DECC [47] and Kim et al. [48]. The average of all the DFOS temperature data at all depths during curing is plotted against time to build a reference curve (dot data in Figure 9), which represents the temperature changes over an average pile concrete cover or radius. This average pile radius, which is used in the finite element model, is calculated as 735 mm from the volume of casted concrete obtained from the concrete log. Using the model parameters adopted in this study, the computed temperature curve from the FE analysis is plotted against the data as shown in Figure 9. The modeled curve closely matches with the average temperature DFOS data which adds confidence in using the parameters listed in Table 2 and Table 3.

### 4.3. FE Back Analysis Results

**Figure 10:**
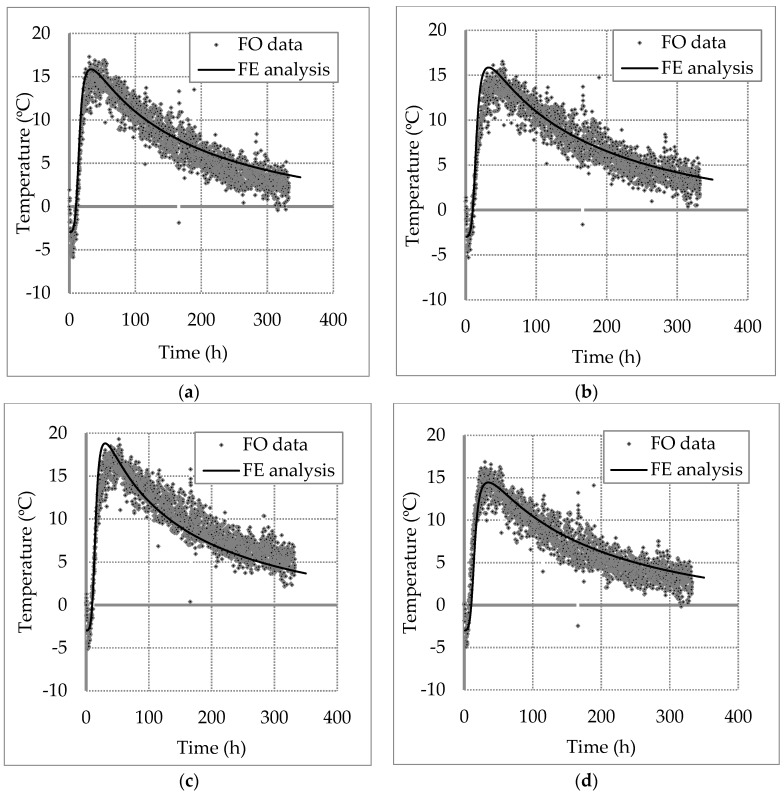
Temperature changes measured with Cable T-1 at different depth: (**a**) 10 m; (**b**) 20 m; (**c**) 30 m; (**d**) 40 m.

The same FE model was used to carry out a series of back analysis to calculate the temperature changes at the four locations over all the cross section spaced at 0.05 m intervals along shaft length. The aim of this back analysis is to find the concrete cover thickness by varying the pile radius of the model (soil/pile interface position) and matching the computed temperature curve to the DFOS data at every given location. For example, the temperature curves of cable T-1 at four different depths are shown in Figure 10. The symbols are the measured temperature data, whereas the solid line is the computed temperature curve that was matched to the data by varying the concrete cover thickness. The trend showed a sharp increase in temperature change over the first two days from −5 °C to about 15–17 °C. After 48 h, the temperature decreased gradually to 5 °C above the initial temperature, indicating that the heat conduction is still occurring.

**Figure 11:**
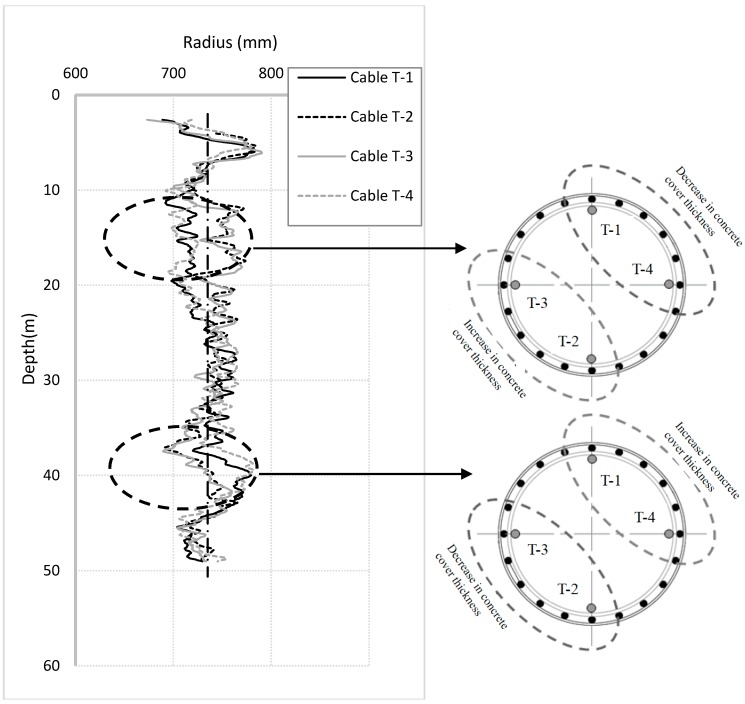
Predicted pile radius in four different axial directions along pile length.

Figure 11 shows the predicted pile radius in the four different axial directions. The results show that notable differences in radius are observed within two regions along the pile. Between 14 and 24 m, the radius at locations of Cables T-4 and T-1 on the northeast half of the pile is 30 mm less than that at locations of cables T-3 and T-2 on the opposite southwest side. Between 35 and 42 m, however, the trend is opposite; the radius on the northeast side is greater than that on the southwest side. This may indicate cage misalignment within these regions.

**Figure 12:**
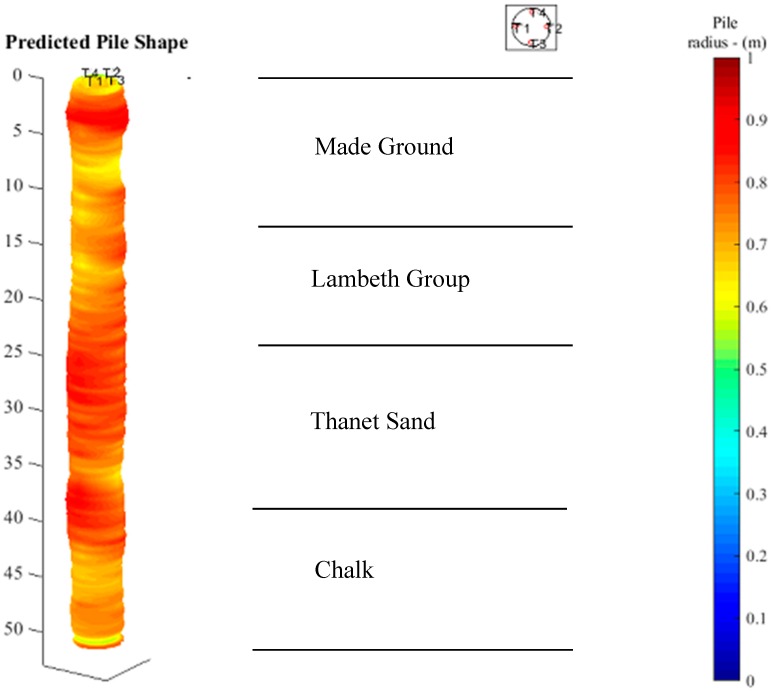
Predicted pile shape.

Figure 12 shows a 3D pile shape according to the predicted radius values discussed above. The radius value between the four axial different directions and longitudinal intervals are calculated by linear extrapolation. The largest pile radius is about 0.78 m and the smallest is about 0.68 m. The red colour indicates an expanded pile radius (larger than the average 0.735 m radius) and the yellow colour a contracted pile radius (smaller than 0.735 m). It shows that the pile radius varies along the pile, especially between 10 and 20 m depth, where it ranges from 0.69 to 0.76 m.

**Figure 13:**
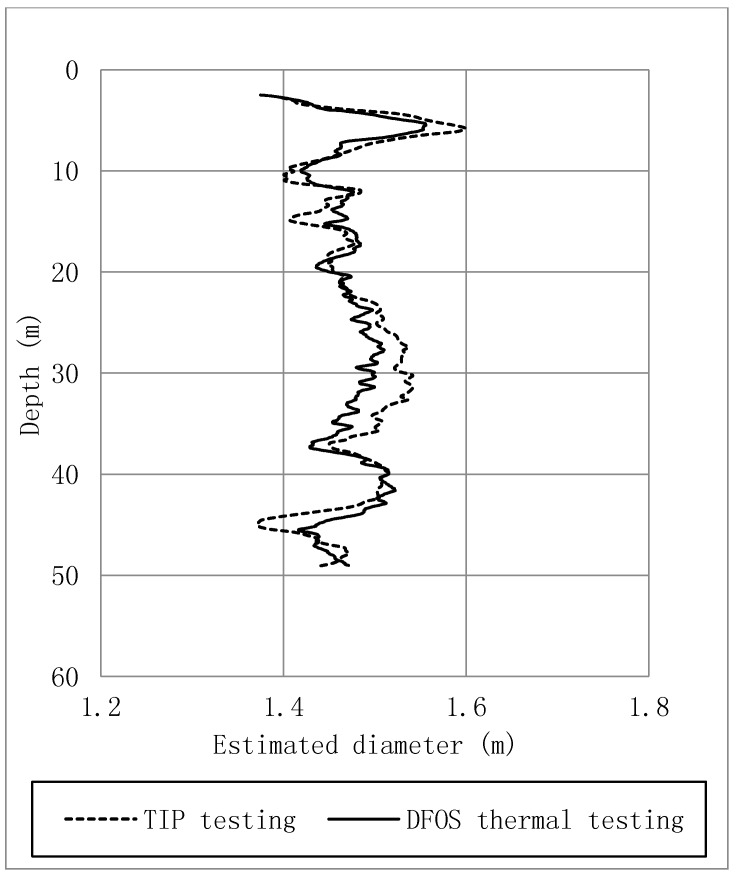
Pile diameter obtained by different test methods.

Thermal integrity profiling (TIP) tests were carried out by means of thermal wire cables which provided another measure of the pile shape for comparison. The effective diameter profile estimated from the proposed thermal integrity testing method (black curve) by averaging the diameter data obtained with the four DFOS cables is close to the values obtained with the three TIP sensor chains (dot curve) 48 h after concrete placement, as shown in Figure 13. The relatively good match between the data from the two testing methods provides confidence in thermal integrity testing using DFOS and FE back analysis. The main difference between TIP and the method used in this paper lies in the measuring and interpretation methods. The differences observed may be due to the influence of a variety of soil thermal properties and thermal boundary conditions, which are not considered in the interpretation of the TIP data.

### 4.4. Impact of Selection of Thermal Properties on the Predicted Pile Radius

The thermal conductivities of soil and concrete vary and hence it is necessary to assess the sensitivity of this thermal property as pile radius is evaluated by conducting back analysis. A series of parametric studies is performed to investigate the effect of thermal conductivity on the performance of the proposed thermal integrity testing. As listed in Table 4, the thermal conductivity values of both soil and concrete are either halved or doubled from the original dataset. The same DFOS temperature data are used but the position of the pile-soil interface (concrete cover thickness) is adjusted to fit the modelled temperature values to the measured data. For a given set of thermal conductivity values, the analysis starts by finding the optimized parameters used in the hydration model as described in Section 3.2 and the values for the model are given in Table 5.

**Figure 14:**
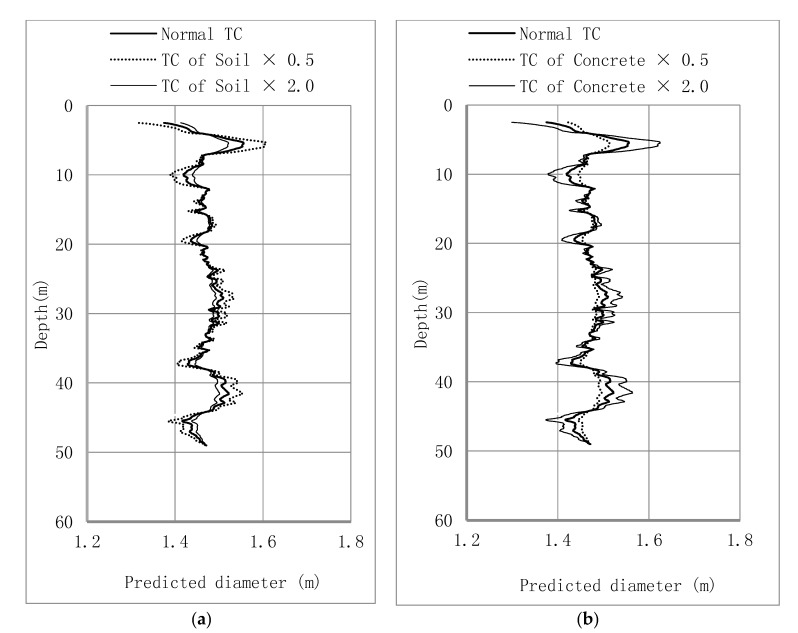
Changes in pile diameter with variation of: (**a**) thermal conductivity of soil; (**b**) thermal conductivity of concrete pile.

In the first parametric study, the thermal conductivity of the soil is varied. As shown in Figure 14a. By decreasing the soil thermal conductivity, the profile of the estimated pile diameter deviates more compared to that estimated from the original data set. Lower thermal conductivity of soil indicates slower heat transfer between the pile and soil, and hence reduce the effects of pile integrity on the diffusion of hydration heat in this problem. Instead, the whole pile heats up with a more uniform temperature distribution along depth. Hence, with the assumed larger fluctuation in pile diameter (the black dot line in Figure 14a, the DFOS still shows the same temperature variation with the other two cases with larger soil thermal conductivity. For this reason, it can be concluded that the temperature cable is less sensitive to the change of concrete cover if the thermal conductivity of soil is lower.

In the second series of parametric study, the thermal conductivity of the pile is varied. As shown in Figure 14b, the effect of pile thermal conductivity is large when analysing the thermal integrity testing data. When concrete has relatively large thermal conductivity, the distribution of temperature within the pile is more uniform and hence DFOS temperature is less sensitive to the change in concrete cover. Therefore, increased thermal conductivity of concrete leads to a larger variety in the pile radius to match the same temperature profile from the in-situ test.

## 5. Conclusions

The need for better integrity testing methods in bored concrete pile construction is evident from a review of literature and industry practice. Thermal integrity testing, which uses the heat generated by hydrating concrete as a measure of integrity, is a promising relatively new technique. This paper proposes the use of DFOS, or other distributed fibre optics method such as DTS, and a simple numerical model as an alternative approach to assess the integrity of the concrete cover of concrete elements. The proposed method was tested on a long pile in the field. The following conclusions are derived:

1. DFOS captures early thermal data very well and is capable of being a standalone system in future installations. The use of temperature as measures of pile integrity shows potential to localise anomalies.
2. The advantages of using DFOS to investigate integrity over other techniques are: (i) its capacity to provides a high spatial density of data and (ii) its ease of installation and (iii) a very low sensor failure rate.
3. Finite element back analysis is able to quantify the relationship between temperature curve and pile radius. The predicted average pile diameter profile is shown to be close to that obtained with the conventional TIP results, providing some validity to the proposed thermal integrity testing and data interpreting method.
4. The selection of thermal conductivity values for concrete and soil can have large effects on evaluating pile profile from DFOS-based temperature curve data. It is therefore necessary to have a good dataset of thermal properties for both concrete and soil in order to ensure the proposed thermal integrity testing method produces good estimate of pile geometry.

## Figures and Tables

**Table 1:** Concrete components.

Material	Type	Weight (Saturated Surface Dry) (kg/m^3^)	Proportion Constituent
Cement CEM II/B-V	CEM I	301	70%
	Pulverised fuedl ash	129	30%
Limestone aggregates	10 to 20 mm	565	32%
	4 to 10 mm	380	21%
	0 to 4 mm	840	47%
Admixture	High-range water reducer	0.75%	--
Water	--	161	--
Total weight		2376	--

**Table 2:** Parameters of hydration model.

Parameters	Q_max_ (J/g)	$q_{\max,20}$ (J/gh)	*a*	*b*	*c*	E (kJ/mol)	R (kJ/mol·K)
Value	35.1	1.01	0.667	3.0	2.6	33.5	0.00831

**Table 3:** Values of thermal properties.

	Thermal Conductivity (W/mK)	Specific Heat Capacity (KJ/m^3^)
Made ground	1.8	2800
Lambeth group	1.6	2400
Thanet sand	1.6	2400
Chalk	1.4	2400
Pile	1.0	2200

**Table 4:** Thermal conductivities for parametric study.

Thermal Conductivity (TC) (W/mK)	Made Ground	Lambeth Group	Thanet Sand	Chalk	Pile
TC of Soil × 0.5	0.9	0.8	0.8	0.7	1.0
TC of Soil × 2	3.6	3.2	3.2	2.8	1.0
TC of Concrete × 0.5	1.8	1.6	1.6	1.4	0.5
TC of Concrete × 2	1.8	1.6	1.6	1.4	2.0

**Table 5:** Parameters of hydration model for parametric study.

Case	Q_max_ (J/g)	q_max,20_ (J/gh)	*a*	*b*	*c*	E (kJ/mol)
TC of Soil × 0.5	30	1.2	0.8	3.0	2.6	33.5
TC of Soil × 2	40	0.8	0.6	3.0	2.6	33.5
TC of Concrete × 0.5	32	0.9	0.667	3.0	2.6	33.5
TC of Concrete × 2	38	1.1	0.667	3.0	2.6	33.5

## References

[1] Ulitskii V.M. (1995). History of pile foundation engineering. Soil Mech. Found. Eng..

[2] Tomlinson M., Woodward J. (2014). Pile Design and Construction Practice.

[3] Bruce D.A., Traylor R.P. (2000). The Repair and Enhancement of Large Diameter Caissons by Grouting. New Technological and Design Developments in Deep Foundations.

[4] Palaneeswaran E., Ramanathan M., Tam C. (2007). Rework in projects: Learning from errors. Surv. Built Environ..

[5] Brown D.A., Turner J.P., Castelli R.J. (2010). Drilled Shafts: Construction Procedures and LRFD Design Methods.

[6] White B., Nagy M., Allin R. Comparing cross-hole sonic logging and low-strain integrity testing results. Proceedings of the Eighth International Conference on the Application of Stress Wave Theory to Piles.

[7] Iskander M., Roy D., Ealy C., Kelley S. (2001). Class-A prediction of construction defects in drilled shafts. Transp. Res. Rec. J. Transp. Res. Board.

[8] Mullins G., Kranc S.C., Johnson K., Stokes M., Winters D. (2007). Thermal integrity testing of drilled shafts. https://www.pile.com/wp-content/uploads/2017/03/FDOT_BD544_20_rpt.pdf.

[9] Mullins G., Winters D. (2011). Infrared Thermal Integrity Testing Quality Assurance Test Method to Detect Drilled Shaft Defects.

[10] (2014). ASTM D7949-14, Standard Test Methods for Thermal Integrity Profiling of Concrete Deep Foundations.

[11] Bungenstab F.C., Beim J.W. (2015). Continousn Flight Auger (CFA) Piles—A Review of the Execution Process and Integrity Evaluation by Low Strain Test. From Fundamentals to Applications in Geotechnics: Proceedings of the 15th Pan-American Conference on Soil Mechanics and Geotechnical Engineering, Buenos Aires, Argentina, 15–18 November 2015.

[12] Piscsalko G., Likins G.E., Mullins G. Drilled Shaft Acceptance Criteria Based Upon Thermal Integrity. Proceedings of the DFI 41st Annual Conference on Deep Foundations.

[13] Cheung L.L.K., Soga K., Bennett P.J., Kobayashi Y., Amatya B., Wright P. (2010). Optical fibre strain measurement for tunnel lining monitoring. Proc. Inst. Civ. Eng. Geotech. Eng..

[14] Mohamad H., Bennett P.J., Soga K., Mair R.J., Bowers K. (2010). Behaviour of an old masonry tunnel due to tunnelling-induced ground settlement. Géotechnique.

[15] De Battista N., Elshafie M.Z.E.B., Soga K., Williamson M.G., Hazelden G., Hsu Y.S. Strain monitoring using embedded distributed fibre optic sensors in a sprayed concrete tunnel lining during the excavation of cross-passages. Proceedings of the 7th International Conference on Structural Health Monitoring of Intelligent Infrastructure.

[16] Bourne-Webb P.J., Amatya B., Soga K., Amis T., Davidson C., Payne P. (2009). Energy pile test at Lambeth College, London: Geotechnical and thermodynamic aspects of pile response to heat cycles. Géotechnique.

[17] Bourne-Webb P.J., Bodas Freitas T.M., Freitas Assunção R.M. (2015). Soil–pile thermal interactions in energy foundations. Géotechnique.

[18] Amatya B.L., Soga K., Bourne-Webb P.J., Amis T., Laloui L. (2012). Thermo-mechanical behaviour of energy piles. Geotechnique.

[19] Mohamad H., Soga K., Amatya B. (2014). Thermal Strain Sensing of Concrete Piles Using Brillouin Optical Time Domain Reflectometry. Geotech. Test. J..

[20] Schwamb T., Soga K., Mair R.J., Elshafie M.Z., Boquet C., Greenwood J. (2014). Fibre optic monitoring of a deep circular excavation. Proc. ICE Geotech. Eng..

[21] Bao X., Chen G. (2016). Temperature-dependent strain and temperature sensitivities of fused silica single mode fiber sensors with pulse pre-pump Brillouin optical time domain analysis. Meas. Sci. Technol..

[22] Bao Y., Hoehler M.S., Smith C.M., Bundy M., Chen G. (2017). Temperature measurement and damage detection in concrete beams exposed to fire using PPP-BOTDA based fiber optic sensors. Smart Mater. Struct..

[23] Pelecanos L., Soga K., Chunge M.P., Ouyang Y., Kwan V., Kechavarzi C., Nicholson D. (2017). Distributed fibre-optic monitoring of an Osterberg-cell pile test in London. Géotech. Lett..

[24] Bao X., Chen L. (2011). Recent progress in Brillouin scattering based fiber sensors. Sensors.

[25] Horiguchi T., Kurashima T., Tateda M. (1989). Tensile strain dependence of Brillouin frequency shift in silica optical fibers. Photonics Technol. Lett..

[26] Kurashima T., Horiguchi T., Tateda M. (1990). Thermal effects on the Brillouin frequency shift in jacketed optical silica fibers. Appl. Opt..

[27] Fellay A. (2003). Extreme Temperature Sensing Using Brillouin Scattering in Optical Fibers. Ph.D. Dissertation.

[28] Kechavarzi C., Soga K., De Battista N., Pelecanos L., Elshafie M.Z.E.B., Mair R.L. (2016). Distributed Fibre Optic Strain Sensing for Monitoring Civil Infrastructure.

[29] Horiguchi T., Shimizu K., Kurashima T., Tateda M. (1995). Development of a distributed sensing technique using Brillouin scattering. J. Lightw. Technol..

[30] Ohno H., Naruse H., Kihara M., Shimada A. (2001). Industrial applications of the BOTDR optical fiber strain sensor. Opt. Fiber Technol..

[31] Zhang H., Wu Z. (2008). Performance evaluation of BOTDR-based distributed fiber optic sensors for crack monitoring. Struct. Health Monit..

[32] Su H., Li J., Hu J., Wen Z. (2013). Analysis and Back-Analysis for Temperature Field of Concrete Arch Dam During Construction Period Based on Temperature Data Measured by DTS. IEEE Sens. J..

[33] Shi N., Chen Y., Li Z. (2016). Crack Risk Evaluation of Early Age Concrete Based on the Distributed Optical Fiber Temperature Sensing. Adv. Mater. Sci. Eng..

[34] Hausner M.B., Suárez F., Glander K.E., Van de Giesen N., Selker J.S., Tyler S.W. (2011). Calibrating single-ended fiber-optic Raman spectra distributed temperature sensing data. Sensors.

[35] Mullins G. (2010). Thermal Integrity Profiling of Drilled Shafts. Deep Found. Inst. J..

[36] Wang X., Ye J., Wang Y. (2008). Hydration mechanism of a novel PCCP+ DCPA cement system. J. Mater. Sci. Mater. Med..

[37] Xu Q., Ruiz J.M., Hu J., Wang K., Rasmussen R.O. (2011). Modeling hydration properties and temperature developments of early-age concrete pavement using calorimetry tests. Thermochim. Acta.

[38] Prosen E.J., Brown P.W., Frohnsdorff G., Davis F. (1985). A multichambered microcalorimeter for the investigation of cement hydration. Cem. Concr. Res..

[39] Bentz D.P. (1995). A Three-Dimensional Cement Hydration and Microstructure Program: I. Hydration Rate, Heat of Hydration, and Chemical Shrinkage.

[40] De Schutter G., Taerwe L. (1995). General hydration model for Portland cement and blast furnace slag cement. Cem. Concr. Res..

[41] De Schutter G., Taerwe L. (1996). Degree of hydration-based description of mechanical properties of early age concrete. Mater. Struct..

[42] Pane I., Hansen W. (2005). Investigation of blended cement hydration by isothermal calorimetry and thermal analysis. Cem. Concr. Res..

[43] Gruyaert E., Robeyst N., De Belie N. (2010). Study of the hydration of Portland cement blended with blast-furnace slag by calorimetry and thermogravimetry. J. Therm. Anal. Calorim..

[44] Ruiz J.M., Schindler A.K., Rasmussen R.O., Nelson P.K., Chang G.K. Concrete temperature modeling and strength prediction using maturity concepts in the FHWA HIPERPAV software. Proceedings of the Seventh International Conference on Concrete Pavements. The Use of Concrete in Developing Long-Lasting Pavement Solutions for the 21st Century.

[45] Schindler A.K. (2004). Effect of temperature on hydration of cementitious materials. Mater. J..

[46] Garber D. (2014). Ground Source Heat Pump System Models in an Integrated Building and Ground Energy Simulation Environment. Ph.D. Dissertation.

[47] UK Department of Energy and Climate Change (DECC) (2008). Microgeneration Installation Standard: MIS 3005.

[48] Kim K.H., Jeon S.E., Kim J.K., Yang S. (2003). An experimental study on thermal conductivity of concrete. Cem. Concr. Res..

